# Determination of Differentiating Markers in Coicis Semen From Multi-Sources Based on Structural Similarity Classification Coupled With UPCC-Xevo G2-XS QTOF

**DOI:** 10.3389/fphar.2020.549181

**Published:** 2020-10-16

**Authors:** Ruyi Zhu, Xiaofen Xu, Qiyuan Shan, Kuilong Wang, Gang Cao, Xin Wu

**Affiliations:** Research Center of TCM Processing Engineering, College of Pharmaceutical Sciences, Zhejiang Chinese Medical University, Hangzhou, China

**Keywords:** markers, coicis semen, triglyceride, qualitative and quantitative, MATLAB

## Abstract

Coicis semen, a medicinal food, is derived from the dried and mature seeds of *Coix lacryma-jobi* L. var. *ma-yuen* (Rom.Caill.) Stapf, a member of the Gramineae family. Lipids are its main constituents. Previous literature reported that coicis semen contains twenty triglycerides and twelve diglycerides. However, we identified thirty-five triglycerides, sixteen diglycerides, four monoglycerides, and two sterols under the preoptimized conditions of UPCC-Xevo G2-XS QTOF combined with a personalized TCM database. Furthermore, we successfully determined glycerol trioleate content to evaluate quality differences. Finally, we identified the fatty acid compositions of seven out of nine differential markers *via* Progenesis QI using principal component analysis, orthogonal projection to latent structures–discriminant analysis, and the LipidMaps database. In addition, we applied a software-based classification, a method that was previously developed by our team, to verify and predict structurally similar compounds. Our findings confirmed that UPCC-Xevo G2-XS QTOF combined with software-based group classification could be used as an efficient method for exploring the potential lipid markers of seed medicine.

## Introduction

Common sense dictates that various natural ingredients exist in TCM. However, most reports on the active components of TCM have focused on polysaccharides, alkaloids, and flavonoids. Fatty oils are widely available ingredients of herbs, and the limited attention that they have received may restrict their further development and application. Fatty oils can be obtained as an active ingredient from animals and plants (Liu et al., 2015; Chen et al., 2016; Hong et al., 2016). A number of TCM contain fatty oils, which are mainly derived from the seeds and fruits of herbs. Diverse fatty oils comprise of glycerol and different types of saturated, monounsaturated, and polyunsaturated fatty acids that each exert therapeutic effects.

Coicis semen (Job’s tears seed or adlay), which has been documented in the 2015 edition of the Chinese pharmacopoeia, is the dry and mature seed of *Coix lacryma-jobi* L. var. *ma-yuen* (Rom.Caill.) Stapf. It is not only a commonly used TCM, but it is also a commonly consumed food. Coicis semen has been proven to have numerous functions, such as detoxification and dampness and arthralgia removal; it also reduces cancer risk (Yu et al., 2011; Bai et al., 2018; Duan, 2018; Xu et al., 2018; Choi et al., 2019; Huang Q. et al., 2019; Huang Y. L. et al., 2019; Li et al., 2019; Liu H. Q. et al., 2019; Liu Y. N. et al., 2019; Manosroi et al., 2019; Qian et al., 2019; Zhang et al., 2019). Moreover, it has a wide range of anti-inflammatory, antioxidation, analgesic, and sedative effects, as well as pharmacological effects against gastric cancer, hepatocellular cancer, Lewis lung cancer, non-small cell lung cancer, pancreatic cancer, and pulmonary cancer (“Fuzheng” category represented by coicis semen oil) (Manosroi et al., 2016a; Manosroi et al., 2016b; Qian et al., 2016; Xi et al., 2016; Wang et al., 2016; Han et al., 2017; Qu et al., 2017a; Schwartzberg et al., 2017; Jang et al., 2018; Kim et al., 2018; Zhang et al., 2018). It has a variety of clinical dosage forms, such as microporous microspheres, microemulsions, and intravenous emulsions (Qu et al., 2016; Qu et al., 2017b; Qu et al., 2017c; Trinh et al., 2017; Chen et al., 2018; Guo et al., 2019). Discrepancies in curative effects can be attributed to the various types, contents, and other nutrients of different fatty oils.

Recent research shows that the variety and content of the active components of different fatty oils remarkably influence human health and have their own specific advantages in treatment. Near-infrared spectroscopy analysis showed that the lipid contents of forty-one polished coicis semen samples range from 5.14% to 9.40%. Coicis semen oils contain seven types of triglycerides (trilinolein, 1,2-linolein-3-olein, 1-palmitin-2-linolein-3-olein, 1-palmitin-2,3-linolein, 1-palmitin-2, 3-olein, triolein, and 1,2-olein-3-linolein). Hou et al. identified twenty triglycerides and twelve diglycerides in the lipid profile of coicis semen and developed a green quantification strategy for simultaneously determining the content of 7 TGs (LLL, LLP, LLO, POL, OOL, OOP, and OOO) by combining core–shell column technology and SSDMCs. Lin et al. found β-sitosterol and stigmasterol in the ethyl acetate fraction of an adlay hull extract. Dong et al. established a rapid and reliable m-SPE approach using magnetic multiwalled carbon nanotubes as the adsorbent for the purification of type A trichothecenes, including T-2 toxins, HT-2 toxins, diacetoxyscirpenol, and neosolaniol, in coicis semen (Dong et al., 2016; Hou et al., 2018a; Hou et al., 2018b; Lin et al., 2019).

However, several components, especially glycerides, of coicis semen oil still require analysis, and the identification of the active ingredients of this material needs in-depth research. Therefore, our team used Acquity UPCC-Xevo G2-XS QTOF coupled with software-based group classification to further excavate, identify, and visually classify active ingredients in coicis semen oils. Coicis semen oils have boundless development prospects and need to be explored in-depth to lay a foundation for its new preparation, development, and extensive clinical application.

## Materials and Methods

### Materials and Reagents

Glycerol trioleate with the purity of more than 99.9% as determined *via* HPLC–ELSD was purchased from the Nature Standard Co. Ltd (Shanghai, China). Acetonitrile and methanol (HPLC–MS grade) were purchased from Merck (Darmstadt, Germany). Ammonium formate (HPLC grade) was purchased from Sigma-Aldrich. High-purity CO_2_ (99.999%) was purchased from the Shanghai Yizhi Industry Gases Co., Ltd. (Shanghai, China). All other reagents used in sample preparation were of analytical grade. Seven batches of dried coicis semen were purchased from different TCM enterprises in China. The manufacturers and batch numbers of the samples were as follows: batch number 180901 (Zhejiang Chinese Medical University Medical Pieces Co., Ltd., Hangzhou); batch number 190101 (Jirentang Pharmaceutical Co., Ltd., Guiyang); batch number 190105 (Zuoli Baicao Herbal Pieces Co., Ltd., Jiangxi); batch number 181201 (Huadong Herbal Pieces Co., Ltd., Hangzhou); batch number 181206 (Zuoli Baicao Herbal Pieces Co., Ltd., Zhejiang); batch number 190122 (Haiyuan Prepared Slices of Chinese Crude Drugs Co., Ltd., Nanjing); and batch number 190216 (Haichang Chinese Medicine Group Co., Ltd., Nanjing).

### Preparation of Reference and Sample Solutions

The appropriate amount of glyceryl trioleate, which was used as the reference substance, was weighed accurately and diluted with n-hexane to prepare a series of working solutions with concentrations of 0.0099, 0.0988, 0.9881, 4.9405, and 9.8810 μg/mL.

All samples were ground and passed through a No. 3 sieve (355 ± 13 μm). The passing rate of the particles was maintained at more than 80%. The sample preparation procedure was as follows:

A total of 50.0 mL of n-hexane was added to 0.6 g of powdered coicis semen. The seed powder was soaked for 2 h and then sonicated (50 kHz, 250 W, KQ-500DB) for 30 min. The supernatant was filtered to obtain the sample solution. The filtrate was diluted with n-hexane 5 times and 100-fold for the analysis of different components of different samples and the quantitative analysis of glyceride trioleate, respectively. A total of 200 μL solution of each batch was mixed together and used as a pooled QC sample solution for the analysis of free fatty acids.

### UPCC-Xevo G2-XS QTOF Parameters

On the basis of preliminary experiments, the final experimental conditions were determined as follows: Liquid phase system: ACQUITY UPCC; column: Torus 2-PIC, 3.0 × 100 mm, 1.7 μm; mobile phase A: CO_2_, mobile phase B: methanol acetonitrile = 9:1; column temperature: 55°C, ABPR: 2600 psi; compensation solution: methanol solution with 0.5 mM ammonium formate; flow rate: 0.5 mL/min; and injection volume: 0.3 μL. The gradient program was used with a flow rate of 1.0 mL/min and was as follows: 0–2 min (1% B), 2–7 min (1%–5% B), 7–10 min (5% B), and 10–13 min (5%–1% B).

The conditions for mass spectrometry are as follows: Mass spectrometry system: Xevo G2-XS QTOF; ionization method: ESI^+/−^; data acquisition mode: MS^E^; MS^E^ impact energy: low, off, high, 20–50 eV; quantitative ion: m/z 902.8177; collection mass range: 100–1200 Da; capillary voltage: 2.0 kV; cone-hole voltage: 40 V; ion source temperature: 120°C; atomization temperature: 500°C; cone-hole gas flow rate: 50 L/h; and atomized gas flow rate: 1000 L/h. Data calibration was performed by using an external reference (LockSpray) with the constant infusion of leucine–enkephalin solution (200 pg/μL) at a flow rate of 5 μL/min.

The data processing software included UNIFI 1.9.4 and Masslynx V 4.1.

### Data Acquisition and Analysis

Xevo G2-XS QTOF uses the patented LockSpray technology to ensure the accuracy of the collected data in real time. High-accuracy mass numbers can be obtained, and the combination of high-accuracy mass numbers and isotope distribution and secondary fragment information accurately provides molecular formulas. Xevo G2-XS QTOF applies patented MS^E^ technology to obtain the primary and secondary mass spectral information of the compounds for further structural confirmation with one injection at the same time.

The ESI^+/−^ mode was used for the analysis of glycerides and free fatty acids. The first step involved understanding the fragmentation pattern of glycerides as a whole. In the second step, by combining the fragmentation patterns and consulting related literature, a UNIFI database for the glyceride analysis of coicis semen was established. In the third step, the self-built database was imported into UNIFI in addition to the ChemSpider online database, and the appropriate analysis method was set. Furthermore, the software automatically analyzed primary and secondary mass spectral information. Finally, it quickly screened out the target through a unique workflow.

Our team developed a classification program in the Visual Basic for Applications (VBA; Microsoft, USA) and MATLAB v7.1 (The Mathworks, Natick, USA) environments. The classification program consisted of three parts (Shan et al., 2012).

A total of 2,916 features were introduced into the SIMCA-P 13.5 software (Umetrics, Umeå, Sweden) for principal component analysis (PCA) and orthogonal projection to latent structures–discriminant analysis (OPLS–DA). The corresponding variable importance in the projection value (VIP value) was calculated in the OPLS–DA model. A potential differential marker was selected when its VIP value exceeded 2.00 and its S-Plot exceeded 0.95.

## Results and Discussion

### Qualitative Results of Glycerides and Free Fatty Acids in Coicis Semen Oils

**Figure 1:**
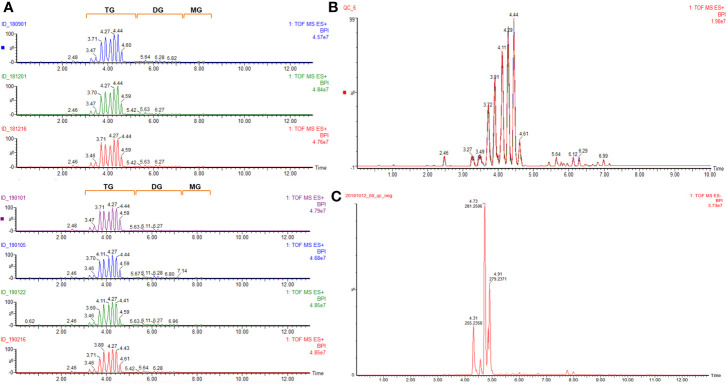
Total ion chromatogram of **(A)** (qualitative analysis of glyceride contents of seven batches of coicis semen oils in ESI^+^); **(B)** (overlay of the QC sample for the differential component analysis of different samples in ESI^+^); **(C)** (qualitative analysis of free fatty acids of QC sample in ESI^−^).

In the ESI^+^ mode, fifty-six and fifty-seven compounds were identified in five (No. 180901, No. 181201, No. 181206, No. 190101, and No. 190122) and two (No. 190105 and No. 190216) batches of samples, respectively. These compounds were mainly composed of glycerides. The total ion chromatogram of all samples is provided in **Figure 1A**, and the corresponding identified glycerides are listed in **Table 1**. Among them, fifty-six common compounds, including thirty-five triglycerides, fifteen diglycerides, four glycerides, and two sterols, were identified in comparison with the corresponding results of twenty triglycerides and twelve diglycerides (Hou et al., 2018a). However, the OP of diglycerides was identified only in two batches of samples, namely 190105 and 190216, likely because of the different processing technologies of different medical enterprises. The QC sample mentioned in **Figure 1B** was overlaid in the following differential component analysis.

**Table 1:** The qualitative results of glycerides in coicis semen oils in ESI^+^.

No.	Component name	Formula	Neutral mass (Da)	Observed m/z	Mass error (mDa)	Observed RT (min)	Response	Adducts
1	β-Sitosterol	C_29_H_50_O	414.3862	*397.3827*	-0.20	2.65	175317	-H2O+H
2	PPP	C_51_H_98_O_6_	806.7363	*824.7724*	2.20	2.87	76543	+NH4, +Na
3	Stigmasterol	C_29_H_48_O	412.3705	*413.3782*	0.40	2.98	280236	+H, +Na, -H2O+H
4	PMO	C_51_H_96_O_6_	804.7207	*822.7572*	2.70	3.01	40426	+NH4, +Na
5	PPS	C_53_H_102_O_6_	834.7676	*852.8045*	3.10	3.11	19271	+NH4, +Na
6	PML	C_51_H_94_O_6_	802.7050	*820.7414*	2.50	3.18	31125	+NH4, +Na
7	PPO	C_53_H_100_O_6_	832.7520	*850.7880*	2.20	3.26	4297765	+NH4, +H, +Na, -H2O+H
8	PSS	C_55_H_106_O_6_	862.7989	*880.8372*	4.40	3.38	5232	+NH4, +Na
9	PPL	C_53_H_98_O_6_	830.7363	*848.7720*	1.90	3.46	4668112	+NH4, +H, +Na, -H2O+H
10	POS	C_55_H_104_O_6_	860.7833	*878.8203*	3.20	3.54	1031215	+NH4, +H, +Na
11	PPOL	C_53_H_96_O_6_	828.7207	*846.7580*	3.40	3.64	344524	+NH4, +H, +Na
12	POO	C_55_H_102_O_6_	858.7676	*876.8048*	3.40	3.71	20321224	+NH4, +H, +Na, -H2O+H
13	OPC20:0	C_57_H_108_O_6_	888.8146	*906.8520*	3.60	3.80	323033	+NH4, +H, +Na
14	LLM	C_53_H_94_O_6_	826.7050	*844.7426*	3.70	3.83	49997	+NH4, +H, +Na
15	PLO	C_55_H_100_O_6_	856.7520	*874.7887*	2.90	3.89	21370394	+NH4, +H, +Na, -H2O+H
16	OOS	C_57_H_106_O_6_	886.7989	*904.8357*	2.90	3.96	3378949	+NH4, +H, +Na, -H2O+H
17	OLC17:0	C_56_H_102_O_6_	870.7676	*888.8046*	3.20	4.01	273530	+NH4, +H, +Na
18	OPC22:0	C_59_H_112_O_6_	916.8459	*934.8839*	4.10	4.03	51651	+NH4, +Na
19	LLP	C_55_H_98_O_6_	854.7363	872.7728	2.60	4.06	8367471	+NH4, +H, +Na, -H2O+H
20	OOO	C_57_H_104_O_6_	884.7833	*902.8202*	3.10	4.11	21932960	+NH4, +H, +Na, -H2O+H
21	OOO20:0	C_59_H_110_O_6_	914.8302	*932.8674*	3.30	4.19	590110	+NH4, +H, +Na
22	OOC19:1	C_58_H_106_O_6_	898.7989	*916.8371*	4.30	4.21	28860	+NH4
23	LLPo	C_55_H_96_O_6_	852.7207	*870.7575*	3.00	4.23	335763	+NH4, +H, +Na, -H2O+H
24	OOL	C_57_H_102_O_6_	882.7676	*900.8044*	2.90	4.27	26267878	+NH4, +H, +Na, -H2O+H
25	OLC20:0	C_59_H_108_O_6_	912.8146	930.8517	3.30	4.34	795167	+NH4, +H, +Na, -H2O+H
26	OLC19:1	C_58_H_104_O_6_	896.7833	*914.8206*	3.40	4.37	39779	+NH4, +Na
27	LLO	C_57_H_100_O_6_	880.7520	*898.7884*	2.60	4.43	22285984	+NH4, +H, +Na, -H2O+H
28	LOC20:1	C_59_H_106_O_6_	910.7989	*928.8353*	2.50	4.51	663937	+NH4, +H, +Na
29	LLC19:1	_C58_H_102_O_6_	894.7676	*912.8009*	-0.60	4.53	34245	+NH4
30	LOC22:0	C_61_H_112_O_6_	940.8459	*958.8826*	2.80	4.54	183383	+NH4, +Na
31	OOO24:0	C_63_H_118_O_6_	970.8928	*988.9309*	4.20	4.57	53885	+NH4
32	LLL	C_57_H_98_O_6_	878.7363	*896.7721*	2.00	4.60	8926747	+NH4, +H, +Na, -H2O+H
33	LLC22:0	C_61_H_110_O_6_	938.8302	*956.8666*	2.60	4.69	163391	+NH4, +Na
34	LOO24:0	C_63_H_116_O_6_	968.8772	*986.9141*	3.10	4.73	113189	+NH4, +Na
35	LLLn	C_57_H_96_O_6_	876.7207	*894.7569*	2.40	4.76	375488	+NH4, +H, +Na, -H2O+H
36	LLC24:0	C_63_H_114_O_6_	966.8615	*984.8983*	2.90	4.87	76278	+NH4, +Na
37	OLS	C_57_H_104_O_6_	884.7833	*885.7856*	-4.90	5.21	3683	+H
38	PP	C_35_H_68_O_5_	568.5067	*551.5035*	0.10	5.43	414186	-H2O+H, +Na
39	PS	C_37_H_72_O_5_	596.5380	*579.5348*	0.10	5.63	579793	-H2O+H, +Na
40	PO	C_37_H_70_O_5_	594.5223	*577.5192*	0.10	5.78	924986	-H2O+H, +H, +Na
41	PL	C_37_H_68_O_5_	592.5067	*575.5031*	-0.30	5.95	728564	-H2O+H, +H, +NH4, +Na
42	OS	C_39_H_74_O_5_	622.5536	*605.5500*	-0.30	5.98	150906	-H2O+H, +H, +Na
43	OO	C_39_H_72_O_5_	620.5380	*603.5344*	-0.30	6.11	2014873	-H2O+H, +H, +NH4, +Na
44	PP	C_35_H_68_O_5_	568.5067	*551.5030*	-0.40	6.19	17291	-H2O+H, +Na
45	OL	C_39_H_70_O_5_	618.5223	*601.5188*	-0.20	6.28	2010772	-H2O+H, +H, +NH4, +Na
46	LL	C_39_H_68_O_5_	616.5067	*617.5130*	-1.00	6.48	681857	+H, +NH4, +Na, -H2O+H
47	OP	C_37_H_70_O_5_	594.5223	*617.5130*	1.40	6.48	681857	+Na, +H, +NH4, -H2O+H
48	LP	C_37_H_68_O_5_	592.5067	*575.5037*	0.40	6.67	311434	-H2O+H, +H, +NH4, +Na
49	OS	C_39_H_74_O_5_	622.5536	*645.5432*	0.30	6.70	44689	+Na, +H, +NH4, -H2O+H
50	OO	C_39_H_72_O_5_	620.5380	*603.5350*	0.30	6.81	707855	-H2O+H, +H, +NH4, +Na
51	LO	C_39_H_70_O_5_	618.5223	*601.5193*	0.20	6.98	697567	-H2O+H, +H, +NH4, +Na
52	LL	C_39_H_68_O_5_	616.5067	*639.4962*	0.30	7.13	505275	+Na, +H, +NH4, -H2O+H
53	LLn	C_39_H_66_O_5_	614.4910	*637.4809*	0.60	7.30	13264	+Na, +H, +NH4, -H2O+H
54	MG(16:0/0:0/0:0)	C_19_H_38_O_4_	330.2770	*353.2667*	0.50	7.97	239151	+Na, -H2O+H
55	MG(17:0/0:0/0:0)	C_20_H_40_O_4_	344.2927	*367.2841*	2.20	8.08	632	+Na, -H2O+H
56	MG(18:0/0:0/0:0)	C_21_H_42_O_4_	358.3083	*381.2977*	0.20	8.19	259875	+Na, -H2O+H
57	MG(20:0/0:0/0:0)	C_23_H_46_O_4_	386.3396	*409.3288*	0.00	8.40	2705	+Na, -H2O+H

A total of thirty free fatty acids were identified in the ESI^−^ mode, and some unsaturated fatty acids could have had isomers, which needed to be confirmed further by using a reference substance. The total ion chromatogram of the QC sample is given in **Figure 1C**, and the corresponding identified free fatty acids are listed in **Table 2**.

**Table 2:** The qualitative results free fatty acids in coicis semen oils in ESI^-^.

No.	Component name	Formula	Neutral mass (Da)	Observed m/z	Mass error (mDa)	Observed RT (min)	Response	Adducts
1	Oleic acid	C_18_H_34_O_2_	282.2559	*281.2486*	0.0	4.74	7887221	-H, +HCOO
2	Linoleic acid	C_18_H_32_O_2_	280.2402	*279.2330*	0.1	4.92	4629367	-H, +HCOO
3	Palmitic acid	C_16_H_32_O_2_	256.2402	*255.2329*	0.0	4.32	2866760	-H, +HCOO
4	Stearic acid	C_18_H_36_O_2_	284.2715	*283.2642*	-0.1	4.58	940078	-H, +HCOO
5	Arachidic acid	C_20_H_40_O_2_	312.3028	*311.2956*	0.0	4.82	131638	-H, +HCOO
6	cis-Vaccenic acid	C_18_H_34_O_2_	282.2559	*281.2484*	-0.2	6.02	126409	-H
7	9, 10- EPOXYOCTADECANOIC ACID	C_18_H_34_O_3_	298.2508	*297.2432*	-0.3	5.79	88969	-H
8	9, 10- EPOXYOCTADECANOIC ACID	C_18_H_34_O_3_	298.2508	*297.2432*	-0.3	5.92	79177	-H
9	11-Eicosenoic acid	C_20_H_38_O_2_	310.2872	*309.2798*	-0.1	4.97	75288	-H
10	Linolenic acid	C_18_H_30_O_2_	278.2246	*277.2172*	-0.1	5.09	61260	-H
11	Lignoceric acid	C_24_H_48_O_2_	368.3654	*367.3579*	-0.2	5.23	58141	-H
12	Behenic acid	C_22_H_44_O_2_	340.3341	*339.3265*	-0.3	5.03	55860	-H
13	Palmitoleic acid	C_16_H_30_O_2_	254.2246	*253.217*3	0.0	4.50	38739	-H
14	Margaric acid	C_17_H_34_O_2_	270.2559	*269.2486*	0.0	4.45	29580	-H
15	Arachidonic acid	C_20_H_32_O_2_	304.2402	*349.2359*	-2.5	4.74	22657	+HCOO
16	TRICOSANOIC ACID	C_23_H_46_O_2_	354.3498	*353.3422*	-0.3	5.13	17977	-H
17	Myristic acid	C_14_H_28_O_2_	228.2089	*227.2017*	0.1	4.05	15915	-H
18	10-Heptadecenoic acid	C_17_H_32_O_2_	268.2402	*267.2327*	-0.3	4.62	11423	-H
19	Eicosapentaenoic acid	C_20_H_30_O_2_	302.2246	*347.2200*	-2.8	4.92	11013	+HCOO, -H
20	Hexacosanoic acid	C_26_H_52_O_2_	396.3967	*395.3887*	-0.7	5.42	8914	-H
21	Pentacosanoic acid	C_25_H_50_O_2_	382.3811	*381.3733*	-0.5	5.33	8708	-H
22	Pentadecylic acid	C_15_H_30_O_2_	242.2246	*241.2173*	0.0	4.20	8677	-H
23	NONADECANOIC ACID	C_19_H_38_O_2_	298.2872	*297.2798*	-0.1	4.69	6204	-H
24	HENEICOSANOIC ACID	C_21_H_42_O_2_	326.3185	*325.3111*	-0.1	4.93	5972	-H
25	Octacosanoic acid	C_28_H_56_O_2_	424.4280	*423.4200*	-0.7	5.60	5361	-H
26	Laurie acid	C_12_H_24_O_2_	200.1776	*199.1704*	0.0	3.71	5056	-H
27	Erucic acid	C_22_H_42_O_2_	338.3185	*337.3115*	0.3	5.19	4770	-H
28	Nervonic acid	C_24_H_46_O_2_	366.3498	*411.3476*	-0.4	6.38	3848	+HCOO
29	11-14-Eicosadienoic acid	C_20_H_36_O_2_	308.2715	*307.2643*	0.1	5.14	2012	-H
30	Homo gamma linolenic acid	C_20_H_34_O_2_	306.2559	*351.2511*	-3.0	4.58	1984	+HCOO

**Figure 2:**
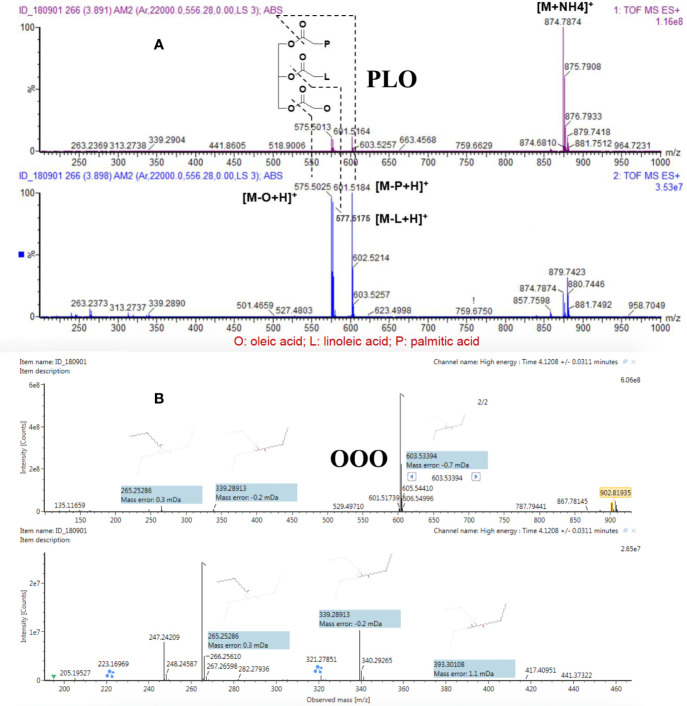
Fragmentation patterns of PLO **(A)** and OOO **(B)** with MS1 and MS2 spectra.

As shown in **Figure 2A**, PLO (t_R_ 3.90 min) provided a precursor ion ([M+NH_4_]^+^) at m/z 874.7874 with a double-bond equivalent. The MS/MS product ions at m/z 601.5184 ([M-P+H]^+^ palmitic acid), m/z 577.5175 ([M-L+H]^+^ linoleic acid), and m/z 575.5025 ([M-O+H]^+^ oleic acid) resulted from the sn-1, sn-2, and sn-3 cleavages of the ester groups, respectively. **Figure 2B** shows the secondary fragment matching diagram for OOO generated by the UNIFI software.

### Software-Based Group Classification of Glycerides

**Figure 3:**
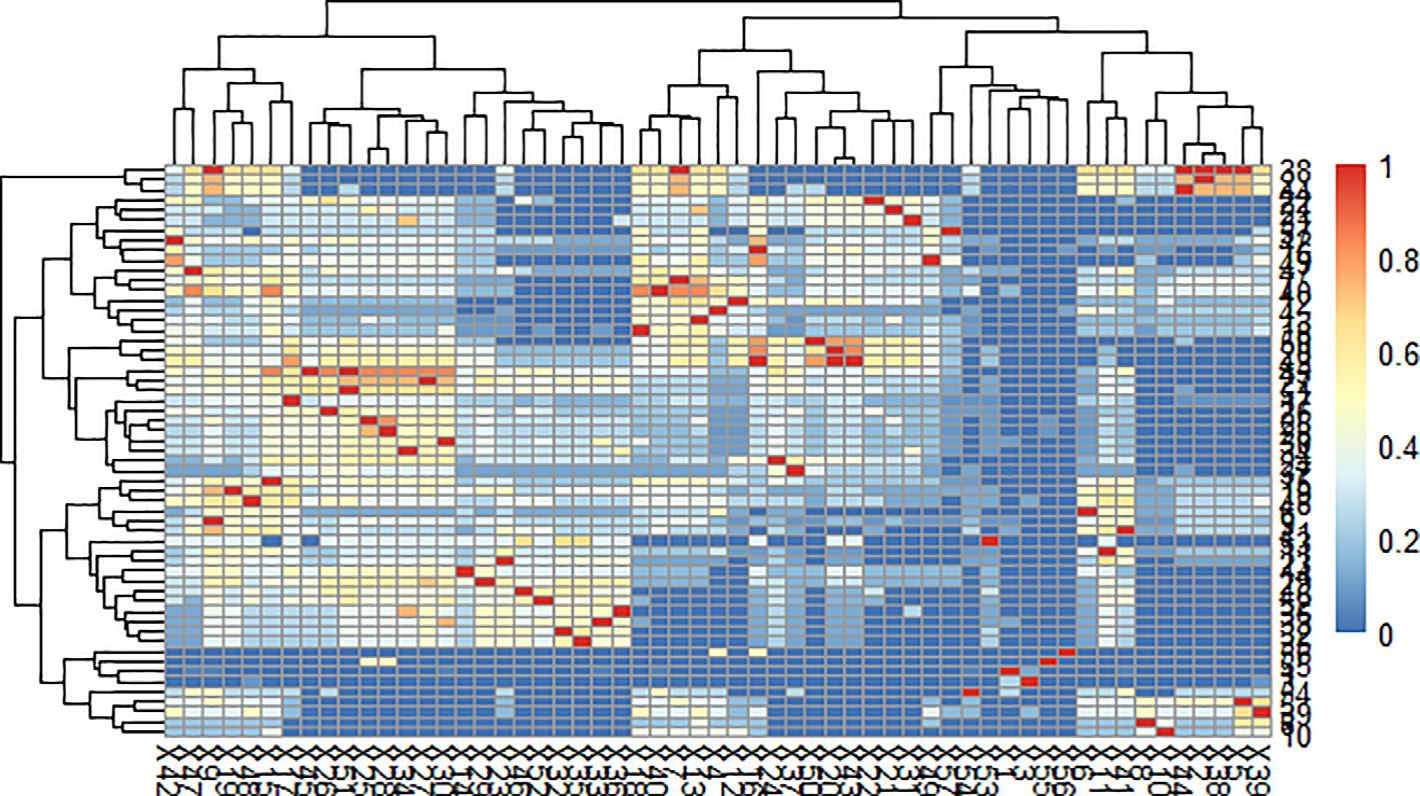
Affinity diagram of the mass spectra of glycerides with software-based group classification.

**Figure 4:**
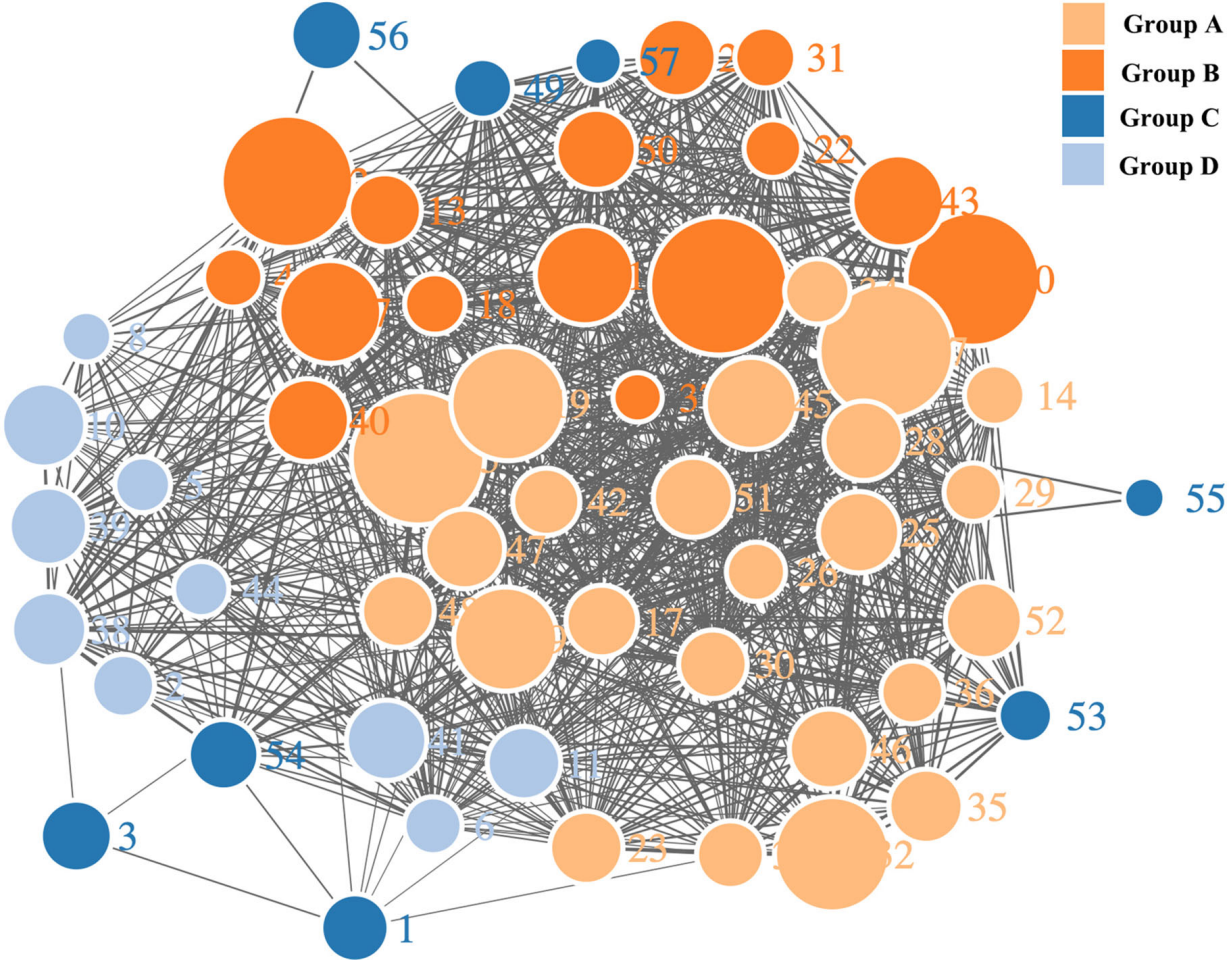
Network diagram of the mass spectra of glycerides with software-based group classification.

Our teams previously developed a program in VBA and MATLAB for the classification of multiple complex components. This program successfully grouped the constituents in the n-hexane extract of coicis semen. Through the comprehensive analysis of herbal samples, fifty-seven peaks were identified and divided into four groups as shown in **Figures 3**, **4**. Three of these groups consisted of triglycerides and diglycerides. The remaining group was composed of four monoglycerides, two diglycerides, and two sterols. The chemical structures and special MS fragmentation pathways of these compounds indicated that the same group might have similar features, and unknown ingredients could be identified through the comprehensive software-based group classification of these compounds.

### Differential Component Analysis of Different Samples

**Figure 5:**
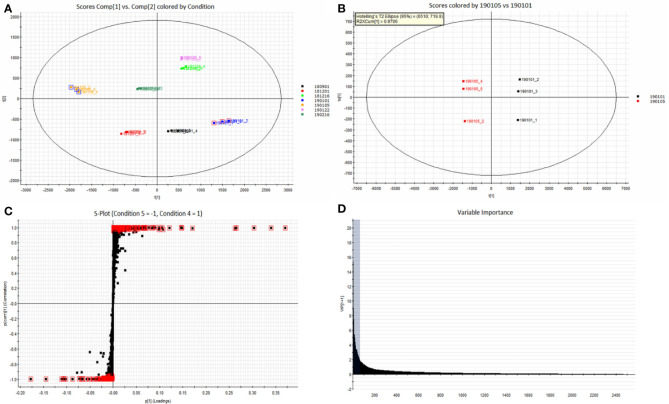
Differential component analysis of different samples. **(A)**: PCA classification of seven batches of samples; **(B)** OPLS–DA analysis of No. 190101 and No. 190105 with significant differences; **(C)** S-Plot of No. 190101 and No. 190105; **(D)** VIP diagram of No. 190101 and No. 190105.

**Figure 6:**
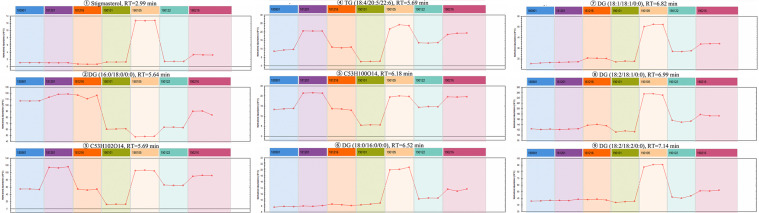
Abundance distribution of nine markers in all samples.

The Progenesis QI omics analysis software was used for differential component research. Before exploring quality markers, the analytical system was first validated for repeatability upon the injection of six QC samples. A total of 2,916 features were extracted and then imported into EZinfo for multivariate statistical analysis. PCA was used to study the variations in the oils of seven batches of coicis semen (**Figure 5A**). The differences between the groups of samples, namely No. 181216 and No.190122, were large. Furthermore, No. 190105 and No. 190101, which showed the largest differences, were subjected to OPLS–DA analysis (**Figure 5B**). These two groups were clearly distinct. Furthermore, we selected compounds with S Plot ≥ 0.95 and VIP ≥ 2 as markers (**Figures 5C, D****)**, and transferred them back to the QI for identification. Finally, nine markers were found (**Table 3**). Then, the LipidBlast, LipidMaps, and Chemspider databases were searched in QI for further identification. Among the nine markers found, seven were identified (five diglycerides, one triglyceride, and one stigmasterol), and the molecular formulas of the remaining two unknown compounds were estimated using an elemental composition tool. The abundance distribution of the nine markers in all the samples is shown in **Figure 6**. The abundance of markers, except diglycerides (16:0/18:0/0:0), in No. 190105 were remarkably higher than that in No. 190101. This result could be related to the largest differences between No. 190105 and No. 190101 and indicated that massive differences in resources and processing technologies existed among medical enterprises.

**Table 3:** The identified results of nine differential markers between No. 190101 and No. 190105.

No.	m/z	Retention time (min)	Identity	Formula	Neutral mass (Da)
1	*413.3781*	2.99	Stigmasterol	C_29_H_48_O	412.3708
2	*579.5337*	5.64	DG(16:0/18:0/0:0)	C_37_H_72_O_5_	596.5370
3	*963.7297*	5.69	unknown	C_53_H_102_O_14_	962.7270
4	*962.7224*	5.69	TG(18:4/20:5/22:6)	C_63_H_92_O_6_	944.6894
5	*961.7175*	6.18	unknown	C_53_H_100_O_14_	960.7113
6	*577.5188*	6.52	DG(18:1/16:0/0:0)	C_37_H_70_O_5_	594.5220
7	*603.5345*	6.82	DG(18:1/18:1/0:0)	C_39_H_72_O_5_	620.5378
8	*641.5118*	6.99	DG(18:2/18:1/0:0)	C_39_H_70_O_5_	618.5222
9	*639.4958*	7.14	DG(18:2/18:2/0:0)	C_39_H_68_O_5_	616.5069

### Quantitation of Glycerol Trioleate in Coicis Semen Oils

#### Investigation of Linear Relations

Reference solutions with concentrations of 0.0099, 0.0988, 0.9881, 4.9405, and 9.8810 μg/mL were used to perform three consecutive injections. The results showed that glyceryl trioleate had a good linear relationship in the range of 0.0090–9.8810 μg/mL, *r^2^*> 0.9990.

#### Quantitative Limit Investigation

The reference solution was diluted stepwise at certain multiples until glyceride trioleate presented S/N ≈ 10. The results showed that the quantitative limit was 4.94 ng/mL.

#### Instrument Precision Inspection

The low, middle, and high concentrations (0.0099, 0.9881, and 9.8810 μg/mL) of the reference solution on the calibration curve were taken and used in six consecutive injections to check instrument precision. The RSD value was less than 3%, which indicated good precision.

#### Repeatability Test

Six powder samples (0.6 g each) of the same batch (No. 180901) were weighed and prepared *via* the sample solution preparation method. The average content of glyceryl trioleate was determined and calculated as 0.91%. The results showed that the RSD value was 4.12% (n = 6).

#### Recovery Rate Test

ine powder samples (0.3 g each) of the same batch of known content were weighed, and then low, medium, and high levels of the three different concentrations of the reference substance were added precisely. The reference substance/sample ratio was controlled at 0.5:1, 1:1, 1.5:1, and each concentration level was tested in triplicate. The results showed a high average recovery of 102.28%.

#### Sample Measurement Results

**Figure 7:**
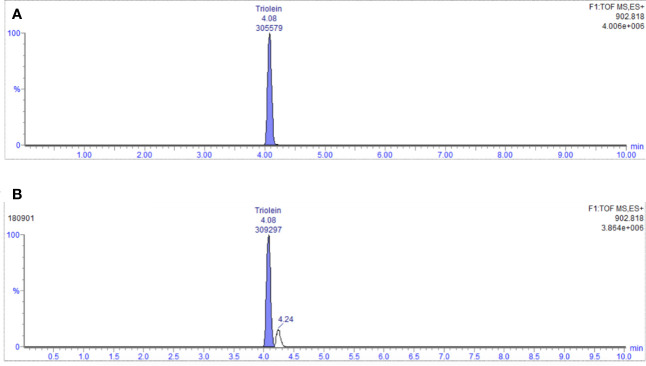
**(A)** Chromatogram of reference (glycerol trioleate); **(B)** EIC diagram of representative sample (No. 180901).

The content of glyceryl trioleate in seven batches of the samples was determined using the established method above. The representative chromatogram is shown in **Figure 7**. Each batch was replicated in triplicate. The average content ranged from 0.84% to 1.05%. The RSD value was less than 5%.

## Conclusion

The established analytical method fully demonstrated that ACQUITY UPCC enabled the fast and efficient chromatographic separation of lipids in coicis semen. Xevo G2-XS QTOF combined with LockSpray real-time external standard mass calibration technology ensured mass accuracy. The data collection method based on MS^E^ tandem mass spectrometry without content discrimination ensured the full collection of information, and one-shot collection could obtain precursor ion and fragment ion information simultaneously with convenient, fast, and high-throughput characteristics.

By using the ACQUITY UPCC/Xevo G2-XS QTOF system combined with the UNIFI software, fifty-seven compounds of glycerides were identified and divided into four groups on the basis of their similar features *via* software-based group classification in the ESI^+^ mode. Moreover, thirty free fatty acids were identified in ESI^−^. In addition, QI omics analysis software found nine differential compounds between No. 190101 and No. 190105, and seven of these compounds were identified. Finally, the quantitative analysis of glyceryl trioleate (quality control component in the 2015 Edition of the Chinese Pharmacopoeia) and methodological verification were performed, and the results showed that the linearity, precision, reproducibility, recovery, and other parameters of the method were good. The established quantitative method determined that the glyceryl trioleate contents of the seven batches of samples ranged from 0.84% to 1.05%.

In summary, we identified additional glycerides and free fatty acids in coicis semen oils. Our results could supplement corresponding component research. Furthermore, nine differential components were found to be potential markers of quality for differentiating coicis semen with different origins. Finally, glyceryl trioleate was determined to evaluate its pros and cons. This approach might be useful for assessing the quality of TCM.

## Data Availability Statement

All datasets presented in this study are included in the article/**Supplementary Material**.

## Author Contributions

Conceptualization: XW and GC. Data curation: RZ, XX, and QS. Formal analysis: KW. Funding acquisition: GC. Investigation: RZ, XX, and KW. Writing—original draft: RZ and XX. Writing—review and editing: XW and GC. All authors contributed to the article and approved the submitted version.

## Funding

This research was supported by the Zhejiang Public Welfare Technology Application Research Project (2017C33175) and the National Natural Science Foundation of China (No. 81703707).

## Conflict of Interest

The authors declare that the research was conducted in the absence of any commercial or financial relationships that could be construed as a potential conflict of interest.
