# Supplementary figures and images for: Determination of Differentiating Markers in Coicis Semen From Multi-Sources Based on Structural Similarity Classification Coupled With UPCC-Xevo G2-XS QTOF
Source: Front Pharmacol. 2020 Oct 16;11:549181. doi: 10.3389/fphar.2020.549181 (PMC7596418; doi:10.3389/fphar.2020.549181)

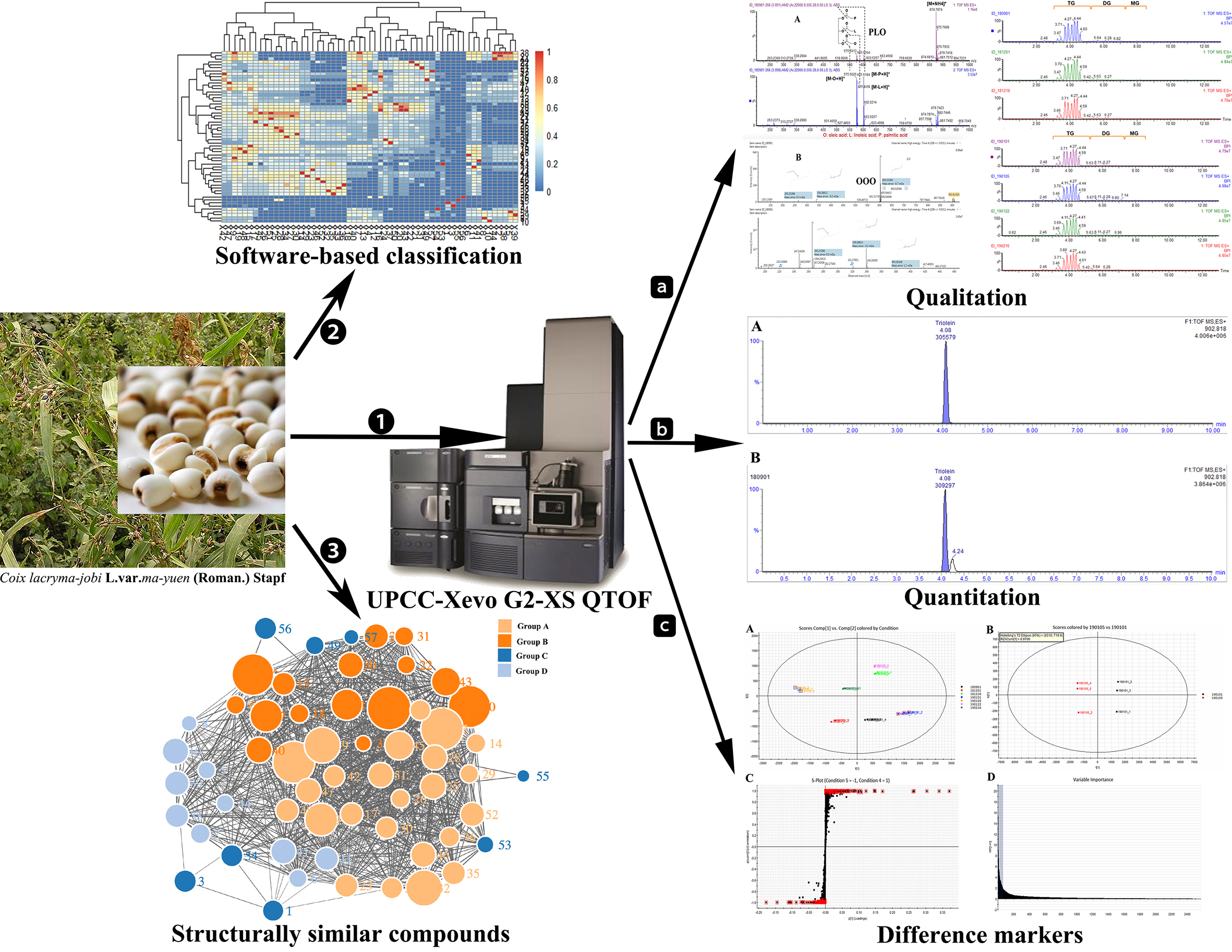

Supplement: Supplementary file 1 [file Image_1.jpeg]
